# Supplementary material for: Music and low-frequency vibrations for the treatment of chronic musculoskeletal pain in elderly: A pilot study
Source: PLoS One. 2021 Nov 2;16(11):e0259394. doi: 10.1371/journal.pone.0259394 (PMC8562790; doi:10.1371/journal.pone.0259394)
Supplement: S2 File — (PDF) [file pone.0259394.s002.pdf]

RESEARCH PROTOCOL

Active Vibration Induced Treatment by Abdominal Excitation (ActiVitae study)

PROTOCOL TITLE Active Vibration Induced Treatment by Abdominal Excitation

**Protocol ID**	NL69608.042.19
**Short title**	ActiVitae
**Version**	3.0
**Date**	04-12-2019
**Coordinating investigator/project leader**	dr R. Soer<br>UMCG Beatrixoord Pijncentrum<br>Dilgtweg 5, 9751 ND, Haren<br>HPC: CD25<br>Postbus: 30.002, 9750 RA, Haren<br>050 36 17424<br>r.soer@umcg.nl
**Principal investigator(s)**<br><br>***Other participating centres:***	Prof dr AP Wolff<br>UMCG Beatrixoord Pijncentrum<br>Dilgtweg 5, 9751 ND, Haren<br>HPC: CD25<br>Postbus: 30.002, 9750 RA, Haren<br>050 36 17424<br>a.p.wolff@umcg.nl<br><br>University Hospital of Copenhagen, Denmark
**Sponsor (in Dutch: verrichter/opdrachtgever)**	University Medical Center Groningen
**Subsidising party**	ZONMW
**Independent expert (s)**<br><br>**Study coordination support**	Dr. J.G.Regtien<br>Head of Department of Critical Care,<br>University Medical Center Groningen<br>Email: j.g.regtien@umcg.nl

PROTOCOL SIGNATURE SHEET

Name	Signature	Date
**Head of Department**<br>Prof. dr. M.M.R.F. Struys		
**Coordinating Investigator/Project leader/Principal Investigator:**<br>Prof. dr A.P. Wolff		

TABLE OF CONTENTS

1. INTRODUCTION AND RATIONALE 10
2. OBJECTIVES 12
3. STUDY DESIGN 13
4. STUDY POPULATION 16
4.1 Population (base) 16
4.2 Inclusion criteria 16
4.3 Exclusion criteria 16
4.4 Sample size calculation 17
5. TREATMENT OF SUBJECTS 18
5.1 Investigational product/treatment 18
5.2 Use of co-intervention (if applicable) 18
5.3 Escape medication (if applicable) 18
6. INVESTIGATIONAL PRODUCT 19
6.1 Name and description of investigational product(s) 19
6.2 Summary of findings from non-clinical studies 22
6.3 Summary of findings from clinical studies 22
6.4 Summary of known and potential risks and benefits 22
6.5 Description and justification of route of administration and dosage 22
6.6 Dosages, dosage modifications and method of administration 22
6.7 Preparation and labelling of Investigational Medicinal Product 22
6.8 Drug accountability 22
7. NON-INVESTIGATIONAL PRODUCT 23
8. METHODS 24
8.1 Study parameters/endpoints 24
8.1.1 Main study parameter/endpoint 24
8.1.2 Secondary study parameters/endpoints (if applicable) 24
8.1.3 Other study parameters (if applicable) 24
8.2 Randomisation, blinding and treatment allocation 24
8.3 Study procedures 24
8.4 Withdrawal of individual subjects 25
8.4.1 Specific criteria for withdrawal (if applicable) 25
8.5 Replacement of individual subjects after withdrawal 25
8.6 Follow-up of subjects withdrawn from treatment 26
8.7 Premature termination of the study 26
9. SAFETY REPORTING 27
9.1 Temporary halt for reasons of subject safety 27
9.2 AEs, SAEs and SUSARs 27
9.2.1 Adverse events (AEs) 27
9.2.2 Serious adverse events (SAEs) 27
9.2.3 Suspected unexpected serious adverse reactions (SUSARs) 28
9.3 Annual safety report 28

9.4 Follow-up of adverse events....28
9.5 Data Safety Monitoring Board (DSMB) / Safety Committee....28
10. STATISTICAL ANALYSIS....29
10.1 Primary study parameter(s)....29
10.2 Secondary study parameter(s)....29
10.3 Other study parameters....29
10.4 Interim analysis....29
11. ETHICAL CONSIDERATIONS....30
11.1 Regulation statement....30
11.2 Recruitment and consent....30
11.3 Objection by minors or incapacitated subjects (if applicable)....30
11.4 Benefits and risks assessment, group relatedness....30
11.5 Compensation for injury....31
11.6 Incentives....31
12. ADMINISTRATIVE ASPECTS, MONITORING AND PUBLICATION....33
12.1 Handling and storage of data and documents....33
12.2 Monitoring and Quality Assurance....33
12.3 Amendments....33
12.4 Annual progress report....33
12.5 Temporary halt and (prematurely) end of study report....33
12.6 Public disclosure and publication policy....34
13. STRUCTURED RISK ANALYSIS....35
13.1 Potential issues of concern....35
13.2 Synthesis....36
14. REFERENCES....40

LIST OF ABBREVIATIONS AND RELEVANT DEFINITIONS

**ABR**	**General Assessment and Registration form (ABR form), the application form that is required for submission to the accredited Ethics Committee; in Dutch: Algemeen Beoordelings- en Registratieformulier (ABR-formulier)**
**AE**	**Adverse Event**
**AR**	**Adverse Reaction**
**ARTS**	**ARthritis Treatment Satisfaction**
**CA**	**Competent Authority**
**CCMO**	**Central Committee on Research Involving Human Subjects; in Dutch: Centrale Commissie Mensgebonden Onderzoek**
**CSI**	**Central Sensitization Index**
**CV**	**Curriculum Vitae**
**DSMB**	**Data Safety Monitoring Board**
**EU**	**European Union**
**EudraCT**	**European drug regulatory affairs Clinical Trials**
**GCP**	**Good Clinical Practice**
**GDPR**	**General Data Protection Regulation; in Dutch: Algemene Verordening Gegevensbescherming (AVG)**
**HADS**	**Hospital Anxiety and Depression Scale**
**HALF-MIS**	**High Amplitude Low Frequency Music Impulse Stimulation**
**IC**	**Informed Consent**
**ICD-10**	**International Statistical Classification of Diseases and Related Health Problems, 10th edition**
**IMDD**	**Investigational Medicinal Device Dossier**
**METC**	**Medical research ethics committee (MREC); in Dutch: medisch-ethische toetsingscommissie (METC)**
**NRS**	**Numeric pain Rating Scale**
**PDI**	**Pain Disability Index**
**QoL**	**Quality of Life**
**QST**	**Quantitative Sensory Testing**
**(S)AE**	**(Serious) Adverse Event**
**SPC**	**Summary of Product Characteristics; in Dutch: officiële productinformatie IB1-tekst**
**Sponsor**	**The sponsor is the party that commissions the organisation or performance of the research, for example a pharmaceutical**

	**company, academic hospital, scientific organisation or investigator. A party that provides funding for a study but does not commission it is not regarded as the sponsor, but referred to as a subsidising party.**
**SUSAR**	**Suspected Unexpected Serious Adverse Reaction**
**UAVG**	**Dutch Act on Implementation of the General Data Protection Regulation; in Dutch: Uitvoeringswet AVG**
**UTAUT**	**Unified Theory of Acceptance and Use of Technology**
**VNS**	**Vagal Nerve Stimulation**
**VAM**	**Vibroacoustic Music Therapy**
**VAT**	**Vibroacoustic Therapy**
**WHO**	**World Health Organisation**
**WMO**	**Medical Research Involving Human Subjects Act; in Dutch: Wet Medisch-wetenschappelijk Onderzoek met Mensen**

SUMMARY

Rationale: Chronic musculoskeletal pain is a disabling condition with huge individual and societal impact in Western society. A considerable amount of patients have decreased functional and biological capacities and lack resilience to stand current treatment standards, including surgery, or medication to handle their pain optimally. Besides, current treatments suffer from side effects. There is a high demand on the development of non-invasive treatments without side effects.

To explain chronic pain syndromes, neuroplasticity has been a point of focus the last decade. Discovering the mechanism of neuroplasticity also suggests the development of new pain relief modalities, that address neuroplasticity and the neuromatrix effectively. Previous studies found that abdominal Pacinian bodies, are related to neuroplasticity via the vagal nerve. Sound impulses below the human ear's frequency (limit about 20 Hz), may be considered as infrasound, activate the Pacinian bodies. Infrasound waves pass without significant attenuation through the air, solid and liquid media. It appears that infrasound with high amplitude affects the human sensory system (via the Pacinian bodies) via Vagal and splanchnic stimulation. Currently, Vagal Nerve Stimulation (VNS) is being used for treatment of resistant cases of eg. pain and depression. Substantial amounts of evidence for this effect have been gathered although the precise mechanism is still unknown. VNS, however, requires an intrusive intervention and has some negative side effects, because of efferent stimulation.

HALF-MIS (High Amplitude Low Frequency Music Impulse Stimulation)', is an innovative treatment modality aimed at stimulation of the Pacinian bodies with the use of infrasound to stimulate vagal activity. Previously, substantial effects were demonstrated on reduction of depression. It is, however, expected to be effective when used in pain management.

Hypotheses: 1. HALF-MIS is a safe and well tolerated intervention. 2. HALF-MIS decreases pain significantly compared to a placebo treatment in elderly patients suffering from chronic musculoskeletal pain. 3. Central sensitization mediates the effect of HALF-MIS.

Study design: Placebo controlled pilot study

Study population: 60 patients (≥65 year) with chronic musculoskeletal pain.

Intervention (if applicable): Each patient will have a schedule consisting of 3 weekly treatments over the course of 3 weeks. Each session will last 20 minutes and 27 seconds according to the length of the music. In all of the eight visits, patients in the treatment group will undergo the HALF-MIS treatment. For this treatment the patient sits relaxed in a chair and gets a belt around the waist with a built-in vibro-tactile unit which delivers vibro-stimulation. For the music patients get a headphone. An iOS app is used to administer the stimuli, synchronize the audio and log the patients' treatments. Patients in the placebo group will follow the same procedure, but without the vibro-stimulation.

Main study parameters/endpoints: Self reported pain (NRS), Quality of life (EuroQol-5D-3L).

Nature and extent of the burden and risks associated with participation, benefit and group relatedness: In total there are 10 visits: 1 screening, 8 treatments and 1 follow-up. During the screening, patients will be asked about their medical history and get a physical examination to determine inclusion. During the first, last en follow-up visits patients have to fill in questionnaires. The first and last treatment visit, patients also undergo quantified sensory testing measurements. Previous trials in depression consistently reported positive effects of treatment and no lasting side effects. In some trials, symptoms such as dizziness and nausea are reported but they are always dose related and pass after the treatment session. Since patients get a pain treatment, the pain intensity might decrease during and/or after the treatments.

1. INTRODUCTION AND RATIONALE

Chronic musculoskeletal pain is a disabling condition with huge individual and societal impact in Western society (1). With an aging society, the relevance for finding suitable and effective treatment modalities also significantly increases for the elderly. These patients frequently suffer from co-morbidities and accompanying poly-pharmacy alongside the chronic pain syndrome. A considerable amount of patients have decreased functional and biological capacities and lack resilience to stand current treatment standards, including surgery, or medication to handle their pain optimally. Besides, current treatments suffer from side effects. There is a high demand on the development of non-invasive treatments without side effects, alleviating pain in elderly and frail patients suffering from musculoskeletal pain.

To explain chronic pain syndromes, the ability of neurostructures to change their reaction when exposed to prolonged stimuli (neuroplasticity) has been a point of focus the last decade. Discovering the mechanism of neuroplasticity also suggests the development of new pain relief modalities, that address neuroplasticity and the neuromatrix effectively (2). Previous studies found that the Pacinian bodies, located in the abdominal cavity, are related to neuroplasticity via the vagal nerve. These bodies are built with free nerve endings protected by multiple keratin layers. While the exact function of these bodies is still ambiguous, the Pacini body frequency response curve is determined by a maximum output at afferent stimulation with a vibration frequency of approx. 240 Hz (3). In principle, however, there is no lower frequency limit as a single stimulation with pressure leading to deformation of the body capsule resulting in an afferent Vagal action potential.

These properties mean that sound impulses below the human ear's frequency limit about 20 Hz (also called infrasound) and activate the Pacinian bodies. Infrasound waves pass without significant attenuation through the air, solid and liquid media. A clarification for this is that the abdominal Pacinian bodies, which perceives the infrasound, are able to register a better signal/noise ratio than the ones located in the skin, as the latter is assumed to be constantly exposed to much higher mechanical stresses from the environment. It appears that infrasound with high amplitude affects the human sensory system (via the Pacinian bodies) via Vagal and splanchnic stimulation, that is why an emotional reaction may be observed. It is our experience that most people react to this with a pleasant sensation. However, a few react with unpleasant sensation, i.e. anxiety evocation. The latter reaction may be reverted by habituation with incremental increase of the low frequency intensity.

Currently, Vagal Nerve Stimulation (VNS) is being used for treatment of resistant cases of pain, depression, epilepsy and migraine attacks (4) by an implanted electronic device and an

electrode attached to the Nervus Vagus. Substantial amounts of evidence for this effect have been gathered although the precise mechanism is still unknown (5). One of the theories proposed has been described in Box 1, below. VNS, however, requires an intrusive intervention and has some negative side effects, because of efferent stimulation (6).

HALF-MIS (High Amplitude Low Frequency Music Impulse Stimulation)', is an innovative treatment modality aimed at stimulation of the Pacinian bodies with the use of infrasound to stimulate vagal activity. Previously, substantial effects were demonstrated on reduction of depression. It is, however, expected to be effective when used in pain management as mental symptoms such as fatigue, depression and cognitive deficits are often prevalent in patients with chronic pain. It is expected that these types of symptoms may be alleviated, as well as somatic pain.

Box 1. Theoretic framework of Pacinian bodies leading to pain reduction
The mesentery and internal organs have high concentrations of Pacinian bodies that are pressure and vibration sensing organs of 0,1 mm in height. The Pacini bodies of the somatosensory system send afferent impulses through the thick myelinated fibers with a synapse before the medulla oblongata. Impulses are routed through a thalamic nucleus (VPL, ventero-postero-lateralis) to the brain's sensory cortex in areas SI and SII. The afferents from the Nervus Vagus reach the Nucleus Tractus Solitarius and are relayed in the VPM (ventero-postero-medialis) of the Thalamus. Impulses from Pacini bodies propagate through the nervous system with (sensory system) maximum amplitude and speed.

The research of Amassian in the 1950ies showed that a simultaneous stimulation of the intestinal nerves (Nn. Splanchnici) and the ulnar nerve resulted in a decrease of the signal in S2 (the parietal lobe, sensory cortex) of the brain – in comparison to ulnar stimulation alone (7). The possibilities of this discovery have never been exploited any further. Later in the century brain mapping research shifted from EEG/EP to functional scanning technologies and brought in-depth knowledge about the cortical area functions and pathways in the brain. Recent studies of the brain's cortical activation by vibrational stimulus have used positron emission tomography and functional MRI to demonstrate a surprising overlap between cortical areas activated by somatic pain, and areas activated by vibration. For instance, SI & SII activation as well as the thalamus, dentate gyrus orbitalis and cingulate (8).

2. OBJECTIVES

Primary Objective:

1. To investigate the tolerability and safety of HALF-MIS used in elderly patients with chronic musculoskeletal pain.
2. To investigate the effect on pain of HALF-MIS used in elderly patients with chronic musculoskeletal pain.
3. To investigate the effect on central sensitization of HALF-MIS used in elderly patients with chronic musculoskeletal pain.

3. STUDY DESIGN

Study design

Placebo controlled pilot study in 60 patients with a chronic musculoskeletal pain syndrome. 30 patients per center will be recruited and screened through one of the participating centers: 1) University Hospital of Copenhagen, Denmark; 2) Anesthesiology Pain Center, University Medical Center Groningen, The Netherlands.The primary examination must result in a cumulated NRS score of more than 3. After randomization there will be a treatment and a control group.

Every patient in the treatment group will receive eight HALF-MIS treatments and the control group eight placebo treatments in three weeks. Every (placebo) treatment will be about 20 minutes and all patients in both groups will undergo all the measurements (NRS, CSI, EuroQol 5D-3L, HADS, PDI, QST, ARTS and UTAUT as described below).
For the recruitment procedure, see paragraph 11.2. The first visit concerns a screening visit for which participants will be invited to the research site at Beatrixoord. When eligible, their treatments will be scheduled. Relevant questionnaires will be sent before each visit. The QST measurements will take place before the first treatment session, after the last treatment session and six weeks after the last treatment session. These items will take about 25-30 minutes to be performed. For an overview of performed questionnaires/measurements see the flowchart at the end of this section.

Treatment

Each patient will have a schedule consisting of 3 weekly treatments over the course of 3 weeks. Each session will last 20 minutes and 27 seconds according to the length of the music. In all of the eight visits, patients in the treatment group will undergo the HALF-MIS treatment. For this treatment the patient have to sit in a chair and get a belt around the waist with a built-in vibro-tactile unit which delivers vibro-stimulation. For the music, patients wear a headphone. An iOS app is used to administer the stimuli, synchronize the audio and log the patients' treatments. Patients in the placebo group will follow the same procedure, but the vibro-stimulation is programmed without any infrasound. The frequencies used are around 240 Hz, that will excite the Pacinian corpuscles in the skin but not penetrate the abdominal wall to elicit a VNS stimulation. In this way, the patient will feel vibrations of the skin, but the actual treatment is not given.

Duration

Each visit will last 45-90 minutes, depending on which questionnaires need to be filled in and whether QST measurements need to take place. Each therapy session will last 20 minutes

and 27 seconds according to the length of the music. Each patient will have a personally fitted treatment schedule consisting of 3 weekly treatments over the course of 3 weeks (8 treatments in total). Including the questionnaires 6 weeks after the final treatment, the duration of this study for the patient will take 9 weeks. Data collection will take 6 months.

Setting

UMCG Anaesthesiology Pain Centre, at Beatrixoord, in Haren

University Hospital of Copenhagen, Denmark

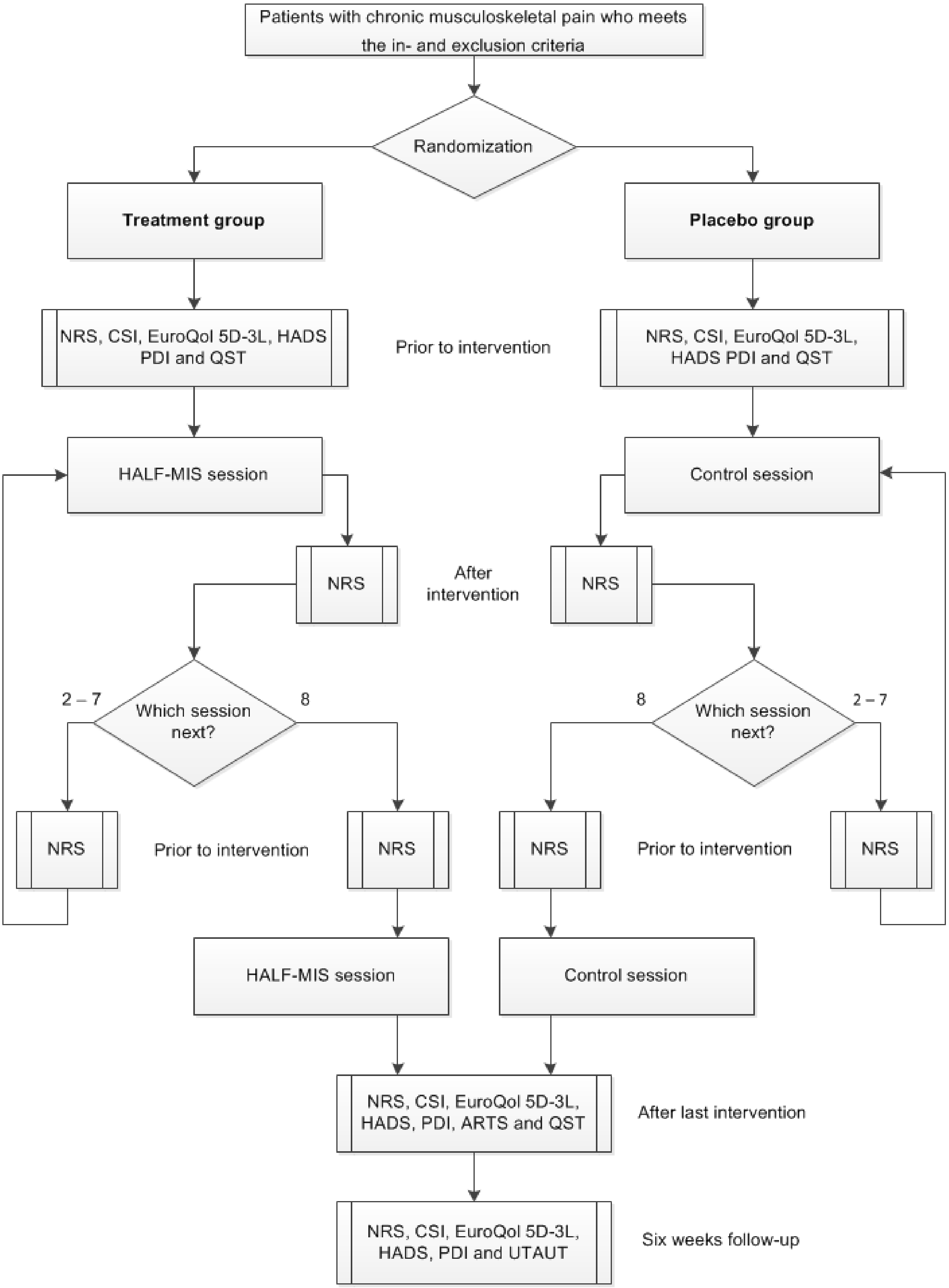
Patients with chronic musculoskeletal pain who meets the in- and exclusion criteria
Randomization
Treatment group
Placebo group
NRS, CSI, EuroQol 5D-3L, HADS PDI and QST
Prior to intervention
NRS, CSI, EuroQol 5D-3L, HADS PDI and QST
HALF-MIS session
Control session
NRS
After intervention
NRS
2 – 7
Which session next?
8
8
Which session next?
2 – 7
NRS
Prior to intervention
NRS
NRS
Prior to intervention
NRS
HALF-MIS session
Control session
NRS, CSI, EuroQol 5D-3L, HADS, PDI, ARTS and QST
After last intervention
NRS, CSI, EuroQol 5D-3L, HADS, PDI and UTAUT
Six weeks follow-up

4. STUDY POPULATION

4.1 Population (base)

The trial will focus on patients with chronic musculoskeletal pain. 60 patients (≥65 year) diagnosed with a chronic musculoskeletal pain syndrome will be recruited through the participating centers. It is meant to include and treat all patients in 6 months. Because of the high prevalence of chronic musculoskeletal pain, the inclusion of 30 patients per participating center shouldn’t be a problem.

4.2 Inclusion criteria

In order to be eligible to participate in this study, a subject must meet all of the following criteria:

1. The symptoms of pain have been present for 3 months or more
2. The symptoms of pain are present every day with a minimum level of NRS=4 (Moderate and severe pain)
3. The symptoms of pain are resulting from a condition diagnosed as a musculoskeletal disease listed in the ICD-10 of the WHO M00-M99.9 (But NOT M50.0, M50.1, M51.0 and M51.1 that are Prolapsus Disci Intervertebralis with myelopathy/radiculopathies – as the pain syndrome in these cases are expected to be predominantly neurogenic)
4. Adequate communication and understanding of the language
5. Age ≥ 65 years
6. Available during the intervention.

4.3 Exclusion criteria

A potential subject who meets any of the following criteria will be excluded from participation in this study:

1. The medical examination showing signs of significant active, untreated psychological comorbidities. Excluded are patients with Delirium and/or psychotic symptoms or moderate or severe depression. The distinction between light depression and moderate and severe depression is done according to the ICD-10: Light depression is F32.0 – moderate and severe depression are F32.1 and F32.2.
2. Patients with decreased signs of autonomic responses, metal implants such as ICD, organ transplantations and surgery on central nervous system in history.
3. The medical examination showing signs of the pain syndrome being exclusively or predominantly neurogenic with the condition diagnosed as a neurological disease listed in the WHO G00-G99.8.

4. The belt doesn’t fit properly.
5. Pain related to malignancies.
6. Enrolled in any other clinical study within the duration of the current study.
7. Enrolled in other music therapy.
8. Incapable of giving consent.

4.4 Sample size calculation

This will be the first trial investigating the effect of Pacinian Vagal Stimulation on pain and quality of life. There is no published or unpublished pilot data on pain available to base a sample size. In this first pilot RCT, we include 30 patients per arm. The outcomes may lead to more insight on the interventional effects, and further RCTs may be developed based on the results.

5. TREATMENT OF SUBJECTS

5.1 Investigational product/treatment

HALF-MIS is primarily a vibratory and auditory stimulus within the range of normal life exposures. As this is a non-drug, non-invasive, non-electrical treatment, NO serious side effects are associated with HALF-MIS.

Similar low frequency exposure has been a part of some music therapy concepts, namely VAT (Vibroacoustic Therapy) and VAM (Vibroacoustic Music Therapy) for decades. In this context low frequency exposure has been investigated (as a black box) in the treatment of various conditions such as pain, depression and spasticity. The trials consistently reported positive effects of treatment and no lasting side effects. In some trials, symptoms such as dizziness and nausea are reported but they are always dose related and pass after the treatment session. We encountered two cases of lens luxation in patients exposed to a shaking chair installation with extremely powerful 5 Hz shaking. This exposure is not considered comparable to the HALF-MIS bass exposure, as it has no content of frequencies below 20 Hz.

5.2 Use of co-intervention (if applicable)

Not applicable.

5.3 Escape medication (if applicable)

Not applicable.

6. INVESTIGATIONAL PRODUCT

6.1 Name and description of investigational product(s)

The RemPulse system consists of a set of components: A controller (currently in the form of an iPad) connected via cables and a standard adapter, the RemPulse app installed on the aforementioned controller, a transducer belt, an ACU and a set of headphones. The patient puts on the transducer belt and headphones, assumes a comfortable position (e.g. reclining or lying down), starts the treatment.

The patient is exposed to vibrations (ca. 20-100Hz, 0-150W) in the abdomen from the transducer belt on their belly, inducing a feeling of comfort in the brain, which, trials suggest, reduces pain and depression symptoms. The 0-150W denotes the power supplied to the transducer and not the actual power induced in the patient’s tissue, which is significantly less. Throughout the treatment session, the patient listens to a relaxing audio track in the headphones; this is harmonically and rhythmically aligned with the aforementioned vibrations. Without the music/said alignment, the vibrations feel quite odd - even disturbing to some - and don’t have the same effect.

Principle of operation

While relaxed by the audio, vibrations are applied on the belly and travel through the skin, spreading out into the abdominal cavity. The vibrations are believed to stimulate the Pacinian Corpuscles, which are present throughout the abdomen. The nerve impulses generated hereby travel through the Vagus Nerve to the brain. Thus, RemPulse is believed to provide VNS.

Features and novelty

RemPulse encompasses the two main features mentioned above:

1. Vibrational abdominal stimulation
2. Relaxing audio

The relaxing audio has no intended therapeutic effect (though it may very well soothe patients with anxiety). Rather, it merely serves to bring the patient to a state of relaxation, where they are receptive to the vibrational treatment. Needless to say, the audio is not novel.

The vibrational abdominal stimulation, however, is a completely novel way to induce what is believed to be VNS (current treatments are invasive/intrusive) and a completely novel way to treat chronic pain.

Accessories and other devices

Essentially, RemPulse consists of an ACU, a transducer belt and a set of headphones. Apart from that, the patient/operator provides an iPad with the RemPulse app installed, which serves as the controller. The iPad is thus an accessory to the system. The cables and adapter connecting the iPad with the ACU are considered part of the ACU for the purpose of classifying accessories.

Configuration and functional elements

Throughout the clinical investigation, RemPulse will only be used in a single hardware configuration without any other variants:

- The ACU is connected to the supply mains through a detachable power supply cord. The appliance coupler is a PowerCON type and will only fit into one socket on the ACU.
- The iPad is connected through an adaptor connecting to the iPad’s Lightning port.
- The adapter is connected to the ACU through an HDMI signal cable and a USB Lightning power supply cable. The former only fits in the only HDMI socket on the ACU and the adapter. The latter only fits in the only USB socket on the ACU; it also connects to the adapter’s Lightning port. It can accidentally also be connected directly to the iPad, in which case the system won’t work; it will just cause the iPad to recharge.
- The headphones are connected to the 3.5mm minijack connector on the iPad, using a detachable cable, the other end of which is connected to the headphones with a proprietary connector. There are no other minijack connectors in the system; neither are there any small, round DC power plugs or similar that could be mistaken for a minijack connector.
- The transducer belt has a fixed cord with a SpeakON connector, which is connected to the only compatible socket in the system.

The RemPulse app is installed on the controller iPad:

1. After installation, the RemPulse app downloads the treatment track, which provides the audio and vibrational stimulation signals. This may take a few minutes, depending on network speed and is only needed once.

2. When the patient is ready (i.e. wearing the belt and headphones and in a relaxed position), the treatment is initiated via the start button on the screen; the button subsequently changes into a stop button. If needed, the treatment can be stopped by pressing the stop button. Otherwise, the treatment continues until the track has finished after about 20 minutes. It is not possible to pause and resume the treatment.
3. Regardless how the treatment ends, the screen again assumes the ready state with a start button, and another treatment can be initiated.
4. The audio volume can be turned up and down as needed using a standard volume slider.
5. The treatment intensity can be adjusted using the + and - buttons between levels 1 through 10, the latter being the highest intensity.

Throughout the clinical investigation, the app may be updated to accommodate any usability issues that emerge. The basic functionality, controls, dosage etc. are not changed.

Functionality

- The iPad administers the signals to the headphones and vibrational transducer, the former of which are connected directly to the iPad. The vibrational signal is sent to the ACU.
- The ACU consists of a digital signal converter, a 280W audio amplifier, a medical isolation transformer and various power components. The digital signal converter changes the digital signal from the iPad into an analogue, low-frequency signal, which is then sent to the amplifier and out to the transducer. All components are powered from the ACU. Mains supply is fed through the isolation transformer, which then powers the amplifier, the signal converter and USB power to the iPad adapter.
- The transducer belt consists of a large belt made from synthetic fabric, which is strapped around the belly and lower back of the patient. On the belt, a mounting plate is affixed on which a vibrational transducer is mounted by the researcher, who received training in standard application of HALF-MIS treatment. The transducer is a 400W (maximum, typical signal is about 100W) 4Ω low-frequency vibrator designed to transmit frequencies between 5Hz and 200Hz. During operation, the mounting plate is roughly centred on the patient’s bellybutton, held tight by the belt. Importantly, the plate must not press against the ribs and hip bones, as this will cause discomfort from the vibrations. Throughout the treatment session, vibrations harmonically and

rhythmically aligned with the audio, are transmitted from the transducer through the belly and into the patients' abdominal cavity.

The headphones provide audio at whatever level is comfortable to the patient and thus function normally.

6.2 Summary of findings from non-clinical studies

Not applicable.

6.3 Summary of findings from clinical studies

We refer to the IMDD (Annex VIII Clinical evidence), pages 48 – 49.

6.4 Summary of known and potential risks and benefits

RemPulse is a prototype using a novel treatment modality. Therefore, a full risk management report does not yet exist. However, RemPulse is a class I medical device, meaning that the risk involved is relatively moderate.

6.5 Description and justification of route of administration and dosage

Not applicable.

6.6 Dosages, dosage modifications and method of administration

We refer to the IMDD (1. Device description and specification), page 6.

6.7 Preparation and labelling of Investigational Medicinal Product

We refer to the IMDD (3. Label(s) and instructions for use), page 9.

6.8 Drug accountability

Not applicable.

7. NON-INVESTIGATIONAL PRODUCT

Not applicable.

8. METHODS

8.1 Study parameters/endpoints

8.1.1 Main study parameter/endpoint per objective

1. Safety and tolerability: (S)AE listings, UTAUT and ARTS
2. Self-reported Pain: NRS
3. Quantitative Sensory Testing: QST

8.1.2 Secondary study parameters/endpoints (if applicable)

1. Not applicable
2. Pain disability index: PDI; Quality of life: EuroQoL-5D-3L
3. Central sensitization index: CSI

8.1.3 Other study parameters (if applicable)

Age, gender, medical history, physical examination, medication use, center and depression and anxiety (HADS).

8.2 Randomisation, blinding and treatment allocation

<u>Randomization</u>

Randomization is secured by the drawing of lots consisting of closed envelopes containing information on whether the patient has been placed in the treatment or the placebo group, as well as an additional description of group definition and content. 30 patients per center are included with a disposition of the lots so that each center will include 15 patients in the treatment group and 15 patients in the control.

<u>Blinding</u>

This will be a double blinded trial. Patients will be blinded for treatment allocation by providing a similar treatment setting but providing a different kind of stimulation. This will create a feeling of vibrations, but lacks the proper frequency to elicit a VNS stimulation. Evaluators will be trained to administer the protocol similarly to both groups. The primary researcher will be blinded to the allocation status of the patients.

8.3 Study procedures

- QST measurements: QST is a psychophysical method that objectively measures responses to calibrated graded innocuous or noxious stimuli and represents, in most respects, an extension of the routine bedside clinical examination of the somatosensory system (3,29). They take 20-25 minutes to be performed.
- CSI: Central sensitization index: Central sensitization symptoms were measured with the CSI. The CSI identifies symptoms related to CS. The CSI consists of 25

questions, each scored 0-4, with a total score range 0-100. Higher scores indicate increased symptom frequency or severity (9).

- NRS (Numeric pain Rating Scale): A pain rating scale were patients rate their pain by giving a number between 0 and 10 (zero = no pain/10 = maximum pain imaginable) (10).
- QoL (EuroQol 5D-3L): The EQ-5D is a six-item questionnaire to investigate QOL. Five questions are categorical (1–3 scale) and one question is on interval level (visual analog scale [VAS] 0–100). The EQ-5D categories measure five dimensions: mobility, self-care, activities of daily life (ADL), pain, and anxiety/depression(10).
- PDI: The Pain Disability Index is a questionnaire that measures the degree to which aspects of the patient's life are disrupted by chronic pain. It lists 7 categories of life activity (family/home responsibilities, recreation, social activity, occupation, sexual behaviour, self-care and life-support activities) (10).
- HADS: The Hospital Anxiety and Depression Scale (HADS) is a self-assessment questionnaire that has been found to be a reliable instrument for detecting states of anxiety and depression in the setting of hospital outpatient clinic. The questionnaire has seven items each for depression and anxiety subscales. Scoring for each item ranges from zero to three, with three denoting highest anxiety or depression level. A total subscale score of >8 points out of a possible 21 denotes considerable symptoms of anxiety or depression. (11)
- ARTS - The ARthritis Treatment Satisfaction (ARTS) questionnaire specifically assesses patients' satisfaction with osteoarthritis treatment. (12,13)
- Pain medication use (self-report)
- Tolerability, acceptability and adoption will be studied using a semi structured interview based on the Unified Theory of Acceptance and Use of Technology (UTAUT). The interview will take place at six weeks follow up.

8.4 Withdrawal of individual subjects

Subjects can leave the study at any time for any reason if they wish to do so without any consequences. The investigator can decide to withdraw a subject from the study for urgent medical reasons.

8.4.1 Specific criteria for withdrawal (if applicable)

Not applicable.

8.5 Replacement of individual subjects after withdrawal

We follow an 'intention to treat' strategy and have no intention to replace subjects after withdrawal.

8.6 Follow-up of subjects withdrawn from treatment

Not applicable.

8.7 Premature termination of the study

Not applicable.

9. SAFETY REPORTING

9.1 Temporary halt for reasons of subject safety

In accordance to section 10, subsection 4, of the WMO, the sponsor will suspend the study if there is sufficient ground that continuation of the study will jeopardise subject health or safety. The sponsor will notify the accredited METC without undue delay of a temporary halt including the reason for such an action. The study will be suspended pending a further positive decision by the accredited METC. The investigator will take care that all subjects are kept informed.

9.2 AEs, SAEs and SUSARs

9.2.1 Adverse events (AEs)

Adverse events are defined as any undesirable experience occurring to a subject during the study, whether or not considered related to the investigational product. All adverse events reported spontaneously by the subject or observed by the investigator or his staff will be recorded.

9.2.2 Serious adverse events (SAEs)

A serious adverse event is any untoward medical occurrence or effect that

- results in death;
- is life threatening (at the time of the event);
- requires hospitalisation or prolongation of existing inpatients' hospitalisation;
- results in persistent or significant disability or incapacity;
- is a congenital anomaly or birth defect; or
- any other important medical event that did not result in any of the outcomes listed above due to medical or surgical intervention but could have been based upon appropriate judgement by the investigator.

An elective hospital admission will not be considered as a serious adverse event.

The investigator will report all SAEs to the sponsor without undue delay after obtaining knowledge of the events.

The sponsor will report the SAEs through the web portal *ToetsingOnline* to the accredited METC that approved the protocol, within 7 days of first knowledge for SAEs that result in death or are life threatening followed by a period of maximum of 8 days to complete the initial preliminary report. All other SAEs will be reported within a

period of maximum 15 days after the sponsor has first knowledge of the serious adverse events.

9.2.3 Suspected unexpected serious adverse reactions (SUSARs)

Not applicable.

9.3 Annual safety report

Not applicable.

9.4 Follow-up of adverse events

All AEs will be followed until they have abated, or until a stable situation has been reached. Depending on the event, follow up may require additional tests or medical procedures as indicated, and/or referral to the general physician or a medical specialist. SAEs need to be reported till end of study within the Netherlands, as defined in the protocol

9.5 Data Safety Monitoring Board (DSMB) / Safety Committee

Not applicable

10. STATISTICAL ANALYSIS

10.1 Primary study parameter(s)

Objective 1: To investigate the tolerability and safety of HALF-MIS used in elderly patients with chronic musculoskeletal pain our primary study parameters are Safety (listing (S)AEs) and tolerability (UTAUT and ARTS). These parameters will be analyzed descriptively.

Objective 2: To investigate the effect on pain of HALF-MIS used in elderly patients with chronic musculoskeletal pain we use one primary endpoint: (self-reported Pain (NRS). Pain intensity will be analysed as appropriate depending on data distribution with a two-sided 0.05 level of significance (superiority in the Half-Miss group). Effect sizes and 95% confidence intervals will be calculated for the primary and secondary outcome measures. Changes within the treatment groups over time as well as differences between groups will be assessed by intention-to-treat analyses. Also the primary analysis will follow the per protocol principle. Sensitivity analyses will be provided to evaluate robustness of the results with regard to centre effects, HADS and/or pain medication.

Objective 3: To investigate the effect on central sensitization of HALF-MIS used in elderly patients with chronic musculoskeletal pain. The primary outcome measure is QST. We will compare outcomes before and after treatment by parametric (students t-test) or non-parametric (Mann-Whitney) tests.

10.2 Secondary study parameter(s)

Secondary endpoints are the PDI and EQ-5D-3L (objective 2) and CSI (objective 3). They will be analyzed exploratory at a two-sided significance level of 5%. The HADS will be used to control for depression as an important confounder, because depression has been hypothesized to change alongside with the chronic pain condition.

10.3 Other study parameters

Descriptive statistics (such as demographics, duration and type of pain and comorbidities): data will be presented as means ± standard deviation or medians ± interquartile ranges.

10.4 Interim analysis

No interim analysis is envisaged for this study.

11. ETHICAL CONSIDERATIONS

11.1 Regulation statement

The study will be conducted in accordance with the principles of the Declaration of Helsinki (14) and in accordance with the Medical Research Involving Human Beings Act (WMO).

11.2 Recruitment and consent

Recruitment occurs on places were the target group is represented, by flyers or by treating physicians mentioning the study to potential participants. In the latter case, the physician can mention the study to potential participants based on information on a ‘physician’ card. When the patient shows interest, the patient will give permission to share contact details with the research team. The ActiVitae physician will inform the potential participant on the study. When still interested, the patient will receive an information letter with informed consent. In the *proefpersooninformatieformulier* (PIF), the aim, nature, duration, adverse effects and potential risks of participation in this investigation is described. Patients can consider their decision for a week and they can withdraw at any moment without consequences for their treatment. When they want to participate, they will sign the informed consent. The screening for suitability for this study, using the in-/exclusion criteria as described in items 4.2 and 4.3, will take place after the informed consent is signed. When the patient is eligible, he/she will be included in the study. For an overview, see the flowchart at the end of this section.

11.3 Objection by minors or incapacitated subjects (if applicable)

Not applicable.

11.4 Benefits and risks assessment, group relatedness

<u>Risk:</u> QST is worldwide applied and considered safe techniques. Since one of the applied stimuli measures pain threshold and pain tolerance, a short-lasting experience of pain might be felt. Risk that a SAE will occur is negligible. Furthermore risks RemPulse is a prototype using a novel treatment modality. Therefore, a full risk management report does not yet exist. However, RemPulse is a class I medical device, meaning that the risk involved is relatively moderate.

<u>Benefit:</u> Since patients get a pain treatment, the pain intensity might decrease during and/or after the treatments.

<u>Group relatedness:</u> not applicable.

11.5 Compensation for injury

The sponsor/investigator has a liability insurance which is in accordance with article 7 of the WMO.

The sponsor (also) has an insurance which is in accordance with the legal requirements in the Netherlands (Article 7 WMO). This insurance provides cover for damage to research subjects through injury or death caused by the study.

The insurance applies to the damage that becomes apparent during the study or within 4 years after the end of the study.

11.6 Incentives

Because patients will get the treatment in their own nursing home, patients don't have to travel. So patients won't get a compensation for travel costs. For the every visit they will receive 5 euros per visit, in total max. €50,-.

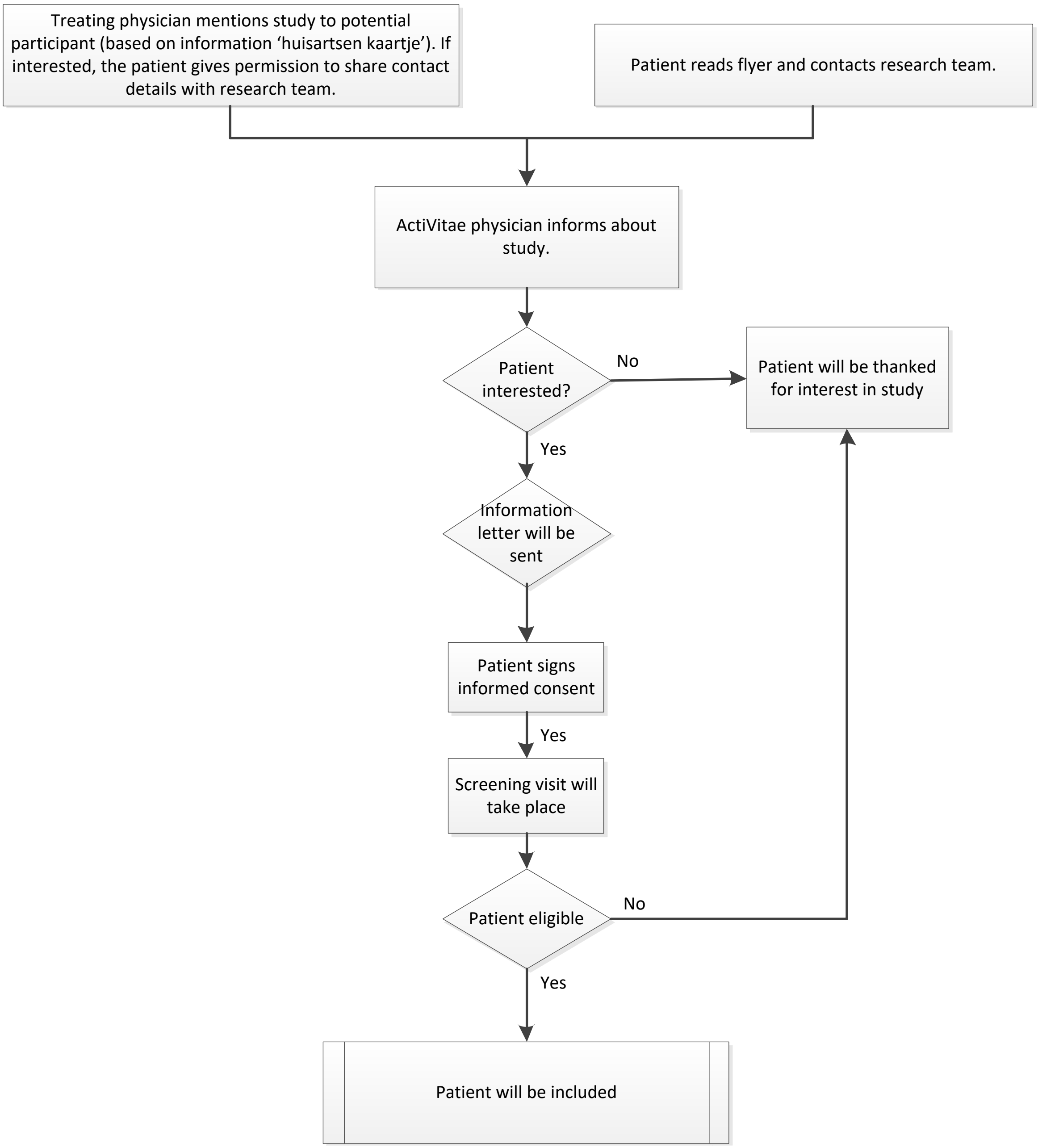
Treating physician mentions study to potential participant (based on information 'huisartsen kaartje'). If interested, the patient gives permission to share contact details with research team.
Patient reads flyer and contacts research team.
ActiVitae physician informs about study.
Patient interested?
No
Patient will be thanked for interest in study
Yes
Information letter will be sent
Patient signs informed consent
Yes
Screening visit will take place
Patient eligible
No
Yes
Patient will be included

12. ADMINISTRATIVE ASPECTS, MONITORING AND PUBLICATION

12.1 Handling and storage of data and documents

Data will be handled confidentially. A subject identification code list will be used to link the data to the subject. The code will not be based on the patient initials and birth date. The key to the code will be safeguarded by the principal investigator.

Data will be accessible for the team of investigators, METC and the *IGJ*.

Data will be saved for 15 years.

Data will/can be used for publication, but no subject will be traceable. The handling of personal data will comply with the Dutch Personal Data Protection Act.

12.2 Monitoring and Quality Assurance

The monitoring is carried out by those responsible for this trial. The research office monitors the trial on a regular basis. This typically includes the following checks:

- The data collected are consistent with adherence to the trial protocol
- CRFs are completed by authorized persons
- No key data are missing
- The data appear to be valid (i.e. range and consistency checks)
- Review of recruitment rates, withdrawals and losses to follow-up

12.3 Amendments

Amendments are changes made to the research after a favourable opinion by the accredited METC has been given. All amendments will be notified to the METC that gave a favourable opinion.

12.4 Annual progress report

The sponsor/investigator will submit a summary of the progress of the trial to the accredited METC once a year. Information will be provided on the date of inclusion of the first subject, numbers of subjects included and numbers of subjects that have completed the trial, serious adverse events/ serious adverse reactions, other problems, and amendments.

12.5 Temporary halt and (prematurely) end of study report

The investigator/sponsor will notify the accredited METC of the end of the study within a period of 8 weeks. The end of the study is defined as the last patient’s last visit.

The sponsor will notify the METC immediately of a temporary halt of the study, including the reason of such an action.

In case the study is ended prematurely, the sponsor will notify the accredited METC within 15 days, including the reasons for the premature termination.

Within one year after the end of the study, the investigator/sponsor will submit a final study report with the results of the study, including any publications/abstracts of the study, to the accredited METC.

12.6 Public disclosure and publication policy

Results of this project will be disclosed unreservedly.

13. STRUCTURED RISK ANALYSIS

13.1 Potential issues of concern

a. Level of knowledge about mechanism of action
See Box 1, section 1.

b. Previous exposure of human beings with the test product(s) and/or products with a similar biological mechanism
The first prototype consisted of a large subwoofer, in front of which the patient was placed. The subwoofer emitted a series of loud booms, which thus induced the abdominal vibrations. This was a rather impractical arrangement, as the noise was not only unpleasant but also annoying to the neighbors. It did, however, yield encouraging results. The current prototype has few similarities with its old predecessor.

After the first subwoofer-based prototype, the next step was to apply the vibrations directly on the patient’s skin using a vibrational transducer, rather than through the air, which was very inefficient and extremely noisy. The first design used a chair, in which the vibrator was built into the lower back, reclining the patient to a comfortable position. The chair prototype is currently being tested clinically in a placebo-controlled depression study.

For simplicity and mobility, another belt-based prototype has been developed, which is the current RemPulse prototype to be used in this project. This version is cheaper and considerably smaller and mobile than the chair version. The prototypes uses the same amplifier and vibration transducer, only the belt applies the vibrations to the belly rather than the lower back. Thus, the effect induced by the prototypes is comparable, and the safety report by Brüel & Kjær of the vibrations from the chair (which apply vibrations relatively directly to the spine) is used as guideline for the belt (which merely applies the vibrations to the belly). It is the intention that, eventually, both prototypes shall result in marketable products.

There are no similar devices on the market today, claiming to provide VNS non-intrusively. Obviously, massage chairs do expose users to vibrations to a certain extent, and music therapy has been known for decades, even with documented effect. Vibratory belts for weight loss also do exist. There are thus a number of different devices on the market with various degrees of overlap with RemPulse but none with the medical therapeutic aim of reducing pain.

In terms of actual VNS, the Vagus Nerve lies rather deeply in the body and cannot easily be stimulated. In order to do so, doctors have so far implanted pacemaker-like devices, which electrically stimulate the Vagus Nerve when activated by remote control. This is a risky procedure involving surgery, and the VNS has numerous side-effects, since the stimulus is electrical and not natural, as is the case with RemPulse, where the normal, physiological mechanism of the Pacinian bodies is exploited. Another VNS treatment has been established, where the skin around the ears is stimulated electrically. A fiber of the Vagus Nerve extends to this area, so by applying sufficient voltage here will induce VNS to some degree. However, as one is only stimulating a small part of the Vagus Nerve, the effect is limited, and applying the needed voltage to the skin is quite uncomfortable and not without risk. RemPulse, on the other hand, is very pleasant, without any significant risk and - preliminary encouraging results suggest - more efficient.

c. Can the primary or secondary mechanism be induced in animals and/or in *ex-vivo* human cell material?
Not applicable.

d. Selectivity of the mechanism to target tissue in animals and/or human beings
Not applicable.

e. Analysis of potential effect
Not applicable.

f. Pharmacokinetic considerations
Not applicable.

g. Study population
See section 4.

h. Interaction with other products
Not applicable.

i. Predictability of effect
Not applicable.

j. Can effects be managed?
Not applicable.

13.2 Synthesis

Hazard	Causes	Risk estimated	Mitigation measures	Risk estimated	Acceptable/not acceptable	Comment	Residual risk, if applicable
**Direct heat exposure from the transducer**	Transducer overheating	Low	No direct skin contact.	Unlikely	Acceptable	Internal self-resetting thermal cut-off further reduces risk. Tests show no alarming surface temperatures, even when overheating.	Unlikely
**Heat exposure from fire**	Transducer or headphones catch fire	Unlikely	No direct skin contact with transducer, plenty of overhead (can withstand 400W), built-in thermal cut-off.		Acceptable	As the transducer is much more powerful than needed and has a built-in thermal cut-off, risk of fire is very low. Furthermore, its cabinet forms a fire enclosure, and it contains no particularly flammable parts. The headphones run on a very weak signal (typically <15mW), so the risk of them actually catching fire is very small. In the unlikely event, they can be removed quickly.	Unlikely
**Excessive vibration caused by failure**	Erroneous, strong signal from ACU	Low	Limited by amplifier	Unlikely	Acceptable	Even in the event of a failure, the safety margin is very large. Any harmful vibrations would have to be endured for many hours on a daily basis to cause harm. Further, the transducer is likely to switch off from overheating long before.	Unlikely

**Excessive vibration caused by overdose**	Overdose	Unlikely	None		Acceptable	As the treatment is well below safety limits, actually using the device to the point of tissue damage is practically impossible and would require using the device all day for many days; it is certainly not possible to do accidentally.	Unlikely
**Risk of electrocution**	Failure	Low	Medical isolation transformer, isolation standoffs etc. to ensure air clearances and creepage distances. Headphones and transducer tested with high voltage to ensure proper insulation.	Unlikely	Acceptable		Unlikely
**Harmful audio levels**	Volume turned up too high, failure	Mid	Audio track is not inherently loud.	Mid	Acceptable	The volume can easily be turned down, and the high volume would have to be endured for some time, before it will cause any harm. In case of failure, headphones can be removed quickly.	Mid, relatively harmless
**Injury**	Dropping transducer onto foot	Low	Neck strap comfortably keeps belt in place while donning/removing belt.	Unlikely	Acceptable		Unlikely
**Harm from malfunctioning software**	Failure or sabotage	Low		Low	Acceptable	The only harm would be strong vibrations (which are only harmful over long periods) or loud audio, in which case the headphones can be removed quickly.	Low, relatively harmless

**Biocompatibility considerations**	Skin allergy	Unlikely		Unlikely	Acceptable	Device only ever has skin contact with patient. The headphones are RoHS compliant, professional studio headphones, CE-approved for the consumer market. The belt is made from medically approved fabric by professional prosthetists.	Unlikely

14. REFERENCES

(1) Hartvigsen J, Hancock MJ, Kongsted A, Louw Q, Ferreira ML, Genevay S, et al. What low back pain is and why we need to pay attention. Lancet 2018 Jun 9;391(10137):2356-2367.

(2) Latremoliere A, Woolf CJ. Central sensitization: a generator of pain hypersensitivity by central neural plasticity. J Pain 2009 Sep;10(9):895-926.

(3) Bolanowski SJ,Jr, Zwislocki JJ. Intensity and frequency characteristics of pacinian corpuscles. I. Action potentials. J Neurophysiol 1984 Apr;51(4):793-811.

(4) Johnson RL, Wilson CG. A review of vagus nerve stimulation as a therapeutic intervention. Journal of inflammation research 2018;11:203-213.

(5) Ben-Menachem E, Revesz D, Simon BJ, Silberstein S. Surgically implanted and non-invasive vagus nerve stimulation: a review of efficacy, safety and tolerability. Eur J Neurol 2015 Sep;22(9):1260-1268.

(6) Chakravarthy K, Chaudhry H, Williams K, Christo PJ. Review of the Uses of Vagal Nerve Stimulation in Chronic Pain Management. Curr Pain Headache Rep 2015 Dec;19(12):6.

(7) Amassian VE. Interaction in the somatovisceral projection system. Res Publ Assoc Res Nerv Ment Dis 1952;30:371-402.

(8) Coghill RC, Talbot JD, Evans AC, Meyer E, Gjedde A, Bushnell MC, et al. Distributed processing of pain and vibration by the human brain. J Neurosci 1994 Jul;14(7):4095-4108.

(9) Kregel J, Vuijk PJ, Descheemaeker F, Keizer D, van der Noord R, Nijs J, et al. The Dutch Central Sensitization Inventory (CSI): Factor Analysis, Discriminative Power, and Test-Retest Reliability. Clin J Pain 2016 Jul;32(7):624-630.

(10) Soer R, Reneman MF, Speijer, Bert L. G. N., Coppes MH, Vroomen, Patrick C. A. J. Clinimetric properties of the EuroQol-5D in patients with chronic low back pain. Spine J 2012 Nov;12(11):1035-1039.

(11) Mayer TG, Neblett R, Cohen H, Howard KJ, Choi YH, Williams MJ, et al. The development and psychometric validation of the central sensitization inventory. Pain Pract 2012 Apr;12(4):276-285.

(12) Pouchot J, Trudeau E, Hellot SC, Meric G, Waeckel A, Goguel J. Development and psychometric validation of a new patient satisfaction instrument: the osteoARthritis Treatment Satisfaction (ARTS) questionnaire. Qual Life Res 2005 Jun;14(5):1387-1399.

(13) Rejas J, Monfort J, Campillo MA, Ruiz MA, Pardo A, Soto J. Criterion validity of the ARthritis Treatment Satisfaction (ARTS) questionnaire: patient satisfaction with treatment and need for switching therapy. Clin Drug Investig 2009;29(8):527-538.

(14) World Medical Association. Declaration of Helsinki - Ethical Principles for Medical Research Involving Human Subjects. 2013; Available at: http://www.wma.net/en/30publications/10policies/b3/. Accessed September 6th, 2015.
