# Supplementary material for: Music and low-frequency vibrations for the treatment of chronic musculoskeletal pain in elderly: A pilot study
Source: PLoS One. 2021 Nov 2;16(11):e0259394. doi: 10.1371/journal.pone.0259394 (PMC8562790; doi:10.1371/journal.pone.0259394)
Supplement: Attachment — Submitted filename: reviewer recommendations -1.docx [file pone.0259394.s004.docx]

Dear Editor,

Thank you for the opportunity to review this article…

Kindly find below my recommendations and suggestions…

Best regards

Abstract:

Conclusion: line 53 the phrase “assumed” does not reflect a conclusive summary… recommend to change to “we believe”

Line 55: trial registration should be removed to the design and methodology section

Introduction

Line 61: you mentioned LBP, neck pain and OA and just gave 2 prevalence (4^th^ and 13^th^)

Line 108: you need to rely on more than 1 reference (38) to support the efficacy of low frequency vibrations

The rationale or research gaps still needs to be more clear

Materials and methods:

Design:

Line 125: As the study was conducted between 125 August 2019 and March 2020, are there any reasons to state publishing your results after 2 years?

~~Line 126: although it is stated that San Martino Hospital (Genova, Italy) participated in the study settings but not of the author affiliations represent it!~~

Patients:

Line 132: what about rheumatic arthritic conditions? Were they excluded?

How was the analgesic effect of medication controlled? Especially if patients were using different medications?

Procedure:

Line 152: full term before acronyms in the first statement

Kindly list who provided the therapy and his/her expertise

Line 180-182: reference for the time and frequency of treatment

Outcomes:

list validity and reliability of the outcome measures used

line 209: has the translation been validated? How?

Statistical analysis:

Line 220: why was the intent to treat used since it’s a pilot study?

Results:

Table 1: kindly add an astrex “*” to the significant value “ weight”

Table 2: p values missing for NRS

Line 304: full term of “AE”

Discussion:

Lines 345-349: are there no other justifications for the non statistical difference rather than the Covid?

The discussion is well written but still lacks consolidated justification of the findings
